# Fever of Unknown Origin: An Unusual Presentation of Kikuchi-Fujimoto Disease

**DOI:** 10.1155/2015/314217

**Published:** 2015-03-22

**Authors:** Piyush Ranjan, Manish Soneja, Nellai Krishnan Subramonian, Vivek Kumar, Shuvadeep Ganguly, Tarun Kumar, Geetika Singh

**Affiliations:** ^1^Department of Medicine, All India Institute of Medical Sciences, New Delhi 110029, India; ^2^All India Institute of Medical Sciences, New Delhi 110029, India; ^3^Department of Pathology, All India Institute of Medical Sciences, New Delhi 110029, India

## Abstract

Kikuchi-Fujimoto disease is a rare, benign, and self-limiting condition that mostly affects young females. Cervical lymphadenopathy with fever is the most common presentation of the disease. It may have unusual presentations that can lead to diagnostic dilemma and delay in diagnosis. We report a case of a 25-year-old female who presented with relapsing fever and cervical lymphadenopathy. Because of atypical presentation, there was a delay in diagnosis and increase in morbidity. High index of suspicion with collaboration between clinicians and pathologists is essential for early and accurate diagnosis of the disease.

## 1. Introduction

Fever of unknown origin (FUO) represents a condition in which the cause of fever remains elusive even after extensive investigations. Infection is the most common cause of FUO, whereas, connective tissue disorders and neoplasms are the other leading causes among adults [1, 2]. Despite considerable development in the imaging and serological and immunohistopathological modalities, the task of diagnosis is often difficult and cannot be achieved in up to 50% of the cases [3]. The diagnosis of FUO becomes even more difficult and often gets delayed when the cause is a rare disease. Kikuchi-Fujimoto disease (KFD) is a rare but important cause of FUO affecting mostly young population. We present an interesting case of FUO in which the diagnosis was delayed due to atypical presentation leading to unnecessary invasive investigations.

## 2. Case Report

A 25-year-old female presented with the complaint of episodes of high grade fever and cervical lymphadenopathy requiring multiple hospitalizations. The patient developed low grade fever with headache three months before for which she was initially prescribed NSAIDS on outpatient basis. Subsequently, she was admitted in an outside hospital for further investigation and management. Her routine hemogram, urine and blood cultures, chest X-ray, and ultrasound of the abdomen were normal. No significant lymphadenopathy was noted. She responded to conservative management and was discharged after defervescence in 4-day time.

After three weeks, she noticed 3-4 small nodular swellings in the neck. She developed high grade fever and headache, following which she was admitted in the hospital. At the time of admission, she had stable vitals and four enlarged, firm, and discrete lymph nodes in the posterior triangle of the neck. There were no meningeal signs. Her routine hemogram showed leukopenia (TLC-3200/*μ*L). Other investigations including urine and blood cultures, a contrast enhanced computed tomography (CECT) of the chest and abdomen along with lumbar puncture, and CSF examination were noncontributory. She was given symptomatic and supportive treatment and the fever subsided and the lymph nodes regressed. She was discharged in two-week time.

**Figure 1:**
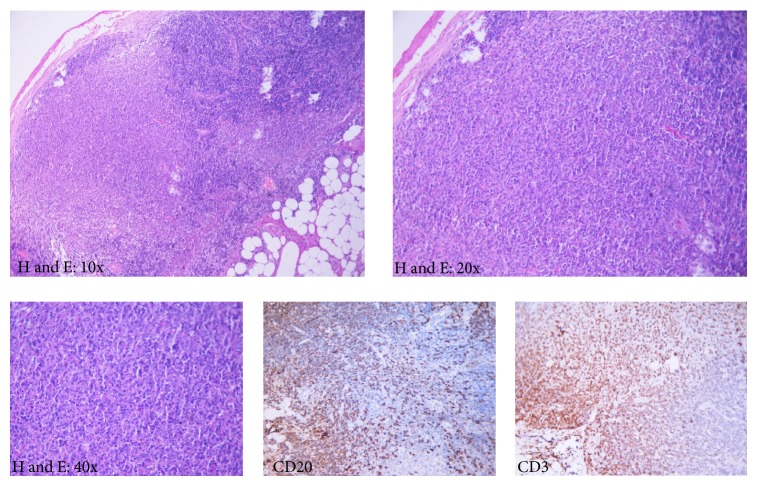
Photomicrophotograph of lymph node biopsy shows paracortical necrosis, histiocytes, apoptotic bodies, and nuclear dust (karyorrhexis). CD3 and CD20 immunostains demonstrate the reactive population of lymphoid cells including both B cells (CD20 immunopositive) and T cells (CD3 immunopositive).

After two weeks, she was again hospitalized with high grade fever and headache. Clinical examination revealed multiple, enlarged, firm, enlargement of cervical and axillary lymph nodes of size about two centimeters. She was again subjected to extensive investigations including lumbar puncture and CSF examination, all of which were noncontributory except for mild anaemia, leukopenia, and raised ESR (70 mm in first hour). Immunological tests like ANA and RF were within normal limits. CECT of chest and abdomen was done and revealed enlarged axillary and cervical lymphadenopathy. Excision biopsy of cervical lymph nodes and histopathological examination was done and revealed multiple well circumscribed areas of necrosis with histiocytes and marked karyorrhexis (Figure 1). These features were suggestive of KFD. To exclude the differential diagnosis of infective aetiology stains for acid fast bacilli and fungus were performed which were negative. The patient was given symptomatic and supportive treatment and was discharged in afebrile condition in one week. She is asymptomatic at 4 months follow-up.

## 3. Discussion

The patient had certain unusual presentation that caused delay in the diagnosis and increase in morbidity. Usually fever is of low to moderate grade and self-limiting in KFD; our case had high grade fever. Cervical lymphadenopathy appeared late in the course and disappeared after first episode of the febrile illness. This probably delayed the decision to do lymph node biopsy. Our case showed early recurrence which is rare in the disease.

KFD is a benign condition of unknown cause that usually presents with lymphadenopathy and fever. It mostly affects younger population with female preponderance. Although more prevalent in Southeast Asia, it is described in different parts of the world including the United States [4].

The etiopathogenesis of KFD is unknown. Current knowledge suggests that it may be due to the excessive immune response of histiocytes to an infectious agent. Many viruses like Epstein Barr virus, human herpes virus, human immunodeficiency virus, parvovirus B19, and paramyxoviruses have been implicated in its pathogenesis [5, 6].

Fever is an important symptom which is present in almost half of the patients [1]. Fever is usually of low grade and persists for weeks. Sometimes, excessive fatigue and joint pains may be present [7]. Lymphadenopathy is present in almost all cases. They are multiple and usually involve cervical group. However other groups like axillary, mediastinal, and inguinal lymph nodes may also be involved. These are usually multiple and moderately enlarged and are typically firm, discrete, and nontender [4]. Other uncommon findings are rash, arthritis, and hepatosplenomegaly that are present in less than 10% of the cases. Complete blood count is usually normal; however cytopenias of various degrees may be present [8]. Immunological antibodies like ANA and RF are usually negative.

The diagnosis of KFD is delayed and, at times, missed mostly because of its rarity and self-limiting course. Lymph node biopsy is the cornerstone of the diagnosis. Histopathological findings of KFD may simulate with that of SLE and lymphoma posing difficulty in diagnosis. There are certain reports where the case was misdiagnosed as lymphoma and cytotoxic drugs were started [4].

The disease has a benign self-limiting course and usually symptomatic and supportive treatment is sufficient. In severe cases with persistent symptoms, high dose steroids are beneficial. In about 10% of the cases, early as well as delayed recurrences have been described [9]. There are reports of the development of SLE in the cases of KFD. Very rarely, death has also been reported [10].

Prolonged pyrexia, lymphadenopathy with noncontributory haematological and biochemical investigations, and sterile cultures of urine and blood usually raise the possibility of relatively common conditions like lymphoma, tuberculosis, SLE, HIV infection, and myeloid tumour. However, KFD is also an important differential in similar settings. High index of suspicion and careful histopathological examination are essential for early and accurate diagnosis. Delayed diagnosis leads to unnecessary investigations and inappropriate aggressive treatment.

This case report shares two important messages. Firstly, KFD can have atypical presentation in the form of high grade fever and late appearance of cervical lymphadenopathy causing delay in the diagnosis. Secondly, there should be low threshold for tissue biopsy as a diagnostic modality in such cases.
